# The kinesin motor protein Kif7 is required for T-cell development and normal MHC expression on thymic epithelial cells (TEC) in the thymus

**DOI:** 10.18632/oncotarget.15241

**Published:** 2017-02-09

**Authors:** Ching-In Lau, Alessandro Barbarulo, Anisha Solanki, José Ignacio Saldaña, Tessa Crompton

**Affiliations:** ^1^ Immunobiology Section, UCL Great Ormond Street Institute of Child Health, London, UK; ^2^ School of Health, Sport and Bioscience, University of East London, London, UK

**Keywords:** Kif7, T-cell development, thymus, thymic epithelial cell, sonic hedgehog, Immunology and Microbiology Section, Immune response, Immunity

## Abstract

Kif7 is a ciliary kinesin motor protein that regulates mammalian Hedgehog pathway activation through influencing structure of the primary cilium. Here we show that Kif7 is required for normal T-cell development, despite the fact that T-cells lack primary cilia. Analysis of Kif7-deficient thymus showed that Kif7-deficiency increases the early CD44+CD25+CD4-CD8- thymocyte progenitor population but reduces differentiation to CD4+CD8+ double positive (DP) cell. At the transition from DP to mature T-cell, Kif7-deficiency selectively delayed maturation to the CD8 lineage. Expression of CD5, which correlates with TCR signal strength, was reduced on DP and mature CD4 and CD8 cells, as a result of thymocyte-intrinsic Kif7-deficiency, and Kif7-deficient T-cells from radiation chimeras activated less efficiently when stimulated with anti-CD3 and anti-CD28 *in vitro*. Kif7-deficient thymocytes showed higher expression of the Hedgehog target gene *Ptch1* than WT, but were less sensitive to treatment with recombinant Shh, and Kif7-deficient T-cell development was refractory to neutralisation of endogenous Hh proteins, indicating that Kif7-deficient thymocytes were unable to interpret changes in the Hedgehog signal. In addition, Kif7-deficiency reduced cell-surface MHCII expression on thymic epithelial cells.

## INTRODUCTION

The thymus is an essential specialised environment for the production of T-cells, in which thymic epithelial cells (TEC) provide cell surface interactions and secreted factors required for T-cell development, including Hedgehog proteins [1–5]. Here we investigate the function in T-cell development of the kinesin motor protein Kif7, a regulatory molecule of the mammalian Hh pathway [6–8].

Kif7 is vertebrate homologue of *Drosophila* Costal 2 (Cos2) [9–11]. In *Drosophila*, Hh signals by binding to its cell surface receptor Patched (Ptch), which then releases the signal transduction molecule Smoothened (Smo) to transduce the signal and activate the transcription factor Ci. Cos2 binds the cytoplasmic tail of Smo and is essential to regulate the activity of Ci. Although many aspects of Hh signalling are highly conserved between *Drosophila* and vertebrates, including the functions of mammalian Ptch1, Smo and the Ci orthologues, Gli1, Gli2 and Gli3, one major difference is that canonical Hh signalling in mammalian cells involves localisation and movement of the signal transduction machinery in the primary cilium [12]. Mammalian Smo has lost its binding site for Kif7 on its cytoplasmic tail, but although initial reports suggested that Kif7 was not involved in Hh signalling in mammalian cells, analysis of Kif7-deficient mice has shown that Kif7 is required to regulate Hh pathway activation, and that it can act as both a positive or negative regulator [7, 8, 11]. Kif7 localizes in the tip of the primary cilium and is believed to regulate Gli activity by controlling cilium structure [6].

In the thymus, Shh promotes TEC differentiation, and mTEC lineage choice [13]. Hh signalling also promotes the earliest stages of T-cell development [5, 14], but negatively regulates pre-TCR induced differentiation from CD4-CD8- double negative [15] to CD4+CD8+ double positive (DP) cell [16, 17], and negatively regulates differentiation from CD4+CD8+ double positive (DP) to mature CD4 single positive (SP) and CD8 SP cell [18–20].

Here we examine the role of Kif7 in T-cell and TEC development in the fetal thymus. T-cells can transduce Hh signals [21], but they lack primary cilia, although they express components of the ciliary transport machinery, which are involved in the immune synapse [22, 23]. It is therefore unclear if Kif7 will be necessary for Hh pathway regulation in the absence of primary cilia in T-cells. Here, we show that Kif7-deficient thymocytes are less sensitive to external modulation of physiological Hh signals than WT thymocytes. We show that in the embryonic thymus Kif7-deficiency increases the CD44+CD25+ DN population, which is the developmental stage at which progenitor cells specify to the T-cell fate. Additionally, Kif7 is required for normal differentiation from DN to DP cell, and influences cell surface CD5 expression, differentiation from DP to mature CD8SP cell, and MHCII-expression by TEC.

## RESULTS

### Kif7 is expressed in the thymus and developing thymocytes

**Figure 1:**
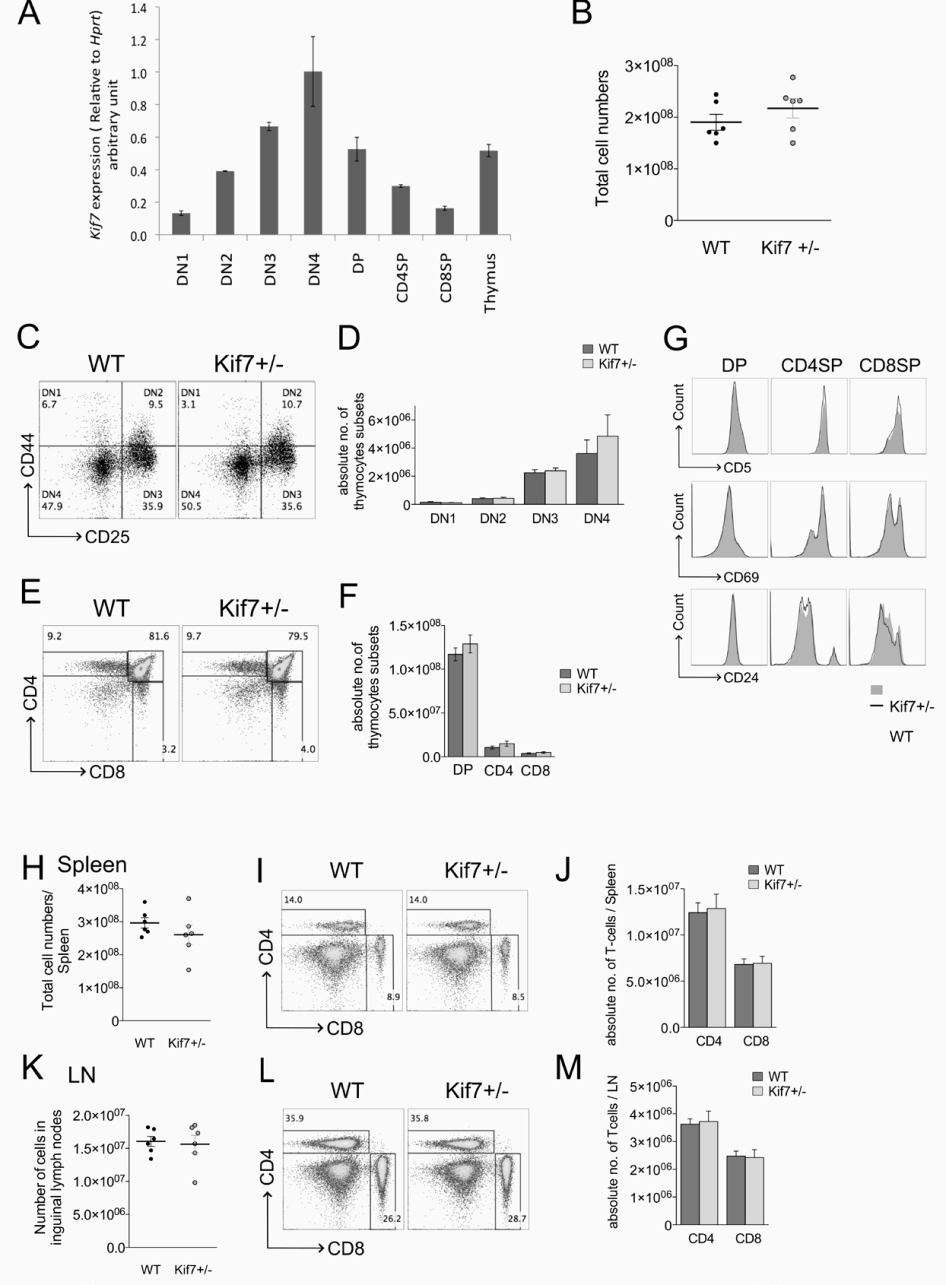
Thymocytes develop normally in Kif7+/− mice In all bar charts in this figure, error bars show the standard error of the mean (SEM). **A**. Bar chart shows *Kif7* transcript levels in FACS sorted DN, DP, SP thymocytes and the whole thymus assessed by quantitative RT-PCR. The expression levels were normalized against *Hprt*. **B**.-**G**. Analysis of developing thymocytes, isolated from WT (*n* = 6) and Kif7+/− (*n* = 6) mice at 6-8 weeks old. **B**. Scatter plot: number of cells in the thymus. Each data point represents a single mouse. The mean for each group is indicated with a line. **C**. Dot plots: flow cytometry profiles of DN subpopulations (DN1-DN4) by surface expression of CD44 and CD25 gated on CD4-CD8- cells. **D**. Mean number of cells in DN1-DN4 subsets in WT and Kif7+/− is shown in bar chart. Percentages of thymocyte subsets (mean+/−SEM): DN1, WT: 2.74+/−0.83 KO:1.75+/−0.34; DN2, WT: 7.46+/−1.72 KO:7.11+/−2.4; DN3, WT: 36.6+/−2.67 KO:34.2+/−3.68; DN4, WT: 53.2+/−4.3 KO:56.9+/−5.65. There were no significant differences in the percentage of thymocyte subsets. **E**. Dot plots: flow cytometry of anti-CD4 and anti-CD8 staining on Kif7+/− and WT thymus, giving percentage of DP, CD4SP, CD8SP cells. **F**. Mean number of cells in DP, CD4SP and CD8SP populations in WT and Kif7+/− thymus is shown in bar chart. Percentages of DP, CD4SP and CD8SP populations (mean+/−SEM): DP, WT: 84.1+/−1.09 KO:81.2+/−0.98; CD4SP, WT: 6.61+/−0.76 KO:8.02+/−0.54; CD8SP, WT: 2.54+/−0.27 KO:2.81+/−0.38. There were no significant differences in the percentage of T-cell populations. **G**. Histograms show cell surface anti-CD5 (upper panel), anti-CD69 (middle panel) and anti-CD24 (lower panel) staining, gated on DP, CD4SP and CD8SP populations in WT and Kif7+/− thymus. **H**.-**J**. Analysis of T-cells in spleen, isolated from WT (*n* = 6) and Kif7+/− (*n* = 6) mice at 6-8 weeks old. **H**. Scatter plot: number of cells in the spleen. Each data point represents a single mouse. The mean for each group is indicated with a line. **I**. Dot plots: flow cytometry of anti-CD4 and anti-CD8 staining on Kif7+/− and WT spleen, giving percentage of CD4 T-cells and CD8 T-cells. **J**. Mean number of cells in CD4 and CD8 T-cell populations in WT and Kif7+/− spleen is shown in bar chart. Percentages of CD4 and CD8 T-cell populations (mean+/−SEM): CD4 T-cell, WT: 14.2+/−1.29 KO:15.2+/−0.79; CD8 T-cell, WT: 7.7+/−0.60 KO:8.19+/−0.45 There were no significant differences in the percentage of T-cell populations. **K**. Scatter plot: number of cells in the two inguinal lymph nodes. Each data point represents a single mouse. The mean for each group is indicated with a line. **L**. Dot plots: flow cytometry of anti-CD4 and anti-CD8 staining on Kif7+/− and WT lymph node, giving percentage of CD4 T-cells and CD8 T-cells. **M**. Mean number of cells in CD4 and CD8 T-cell populations in WT and Kif7+/− LN is shown in bar chart. Percentages of CD4 and CD8 T-cell populations (mean+/−SEM): CD4 T-cell, WT: 37.9+/−1.40 KO:39.6+/−1.51; CD8 T-cell, WT: 25.6+/−0.85 KO:25.4+/−0.63. There were no significant differences in the percentage of T-cell populations.

To investigate the role of Kif7 in the regulation of T-cell development, we analysed *Kif7* expression in whole thymus and facs-sorted adult thymocyte subsets by quantitative(Q) RT-PCR. During thymocyte development, cells pass through well-defined stages: DN cells must rearrange the *TCR*β locus to express the pre-TCR complex, and require pre-TCR signal transduction to differentiate into DP cells. They must then rearrange the *TCR*α locus, and require TCR signal transduction to differentiate from DP to mature CD4SP and CD8SP cell. The DN population can be subdivided by expression to CD44 and CD25. CD44+CD25- (DN1) cells, differentiate to CD44+CD25+ (DN2), then to CD44-CD25+ (DN3), then, after pre-TCR signal transduction to the CD44-CD25- (DN4) population, which progresses to the DP stage, sometimes via an immature CD8+ intermediate (ISP) [4]. We therefore sorted the four DN subsets, in addition to DP, CD4SP and CD8SP populations. Despite the fact that thymocytes lack primary cilia, we detected *Kif7* expression in RNA prepared from all thymocyte subsets throughout T-cell development, as well as the whole thymus. We found relatively low expression in the DN1 population and expression was up-regulated in DN2 and DN3 populations, with peak expression in DN4 cells, and down-regulation in DP and SP populations (Figure 1A).

### T-cells develop normally in the Kif7-heterozygous thymus

Kif7−/− embryos die in utero with severe developmental defects, but Kif7+/− mice develop normally and appear normal [8]. We therefore compared the adult Kif7+/− thymus with Kif7+/+ [24]. The number of thymocytes was similar between Kif7+/− and WT mice (Figure 1B), and we found no significant differences in the proportion of, or number of cells in the DN subsets, DP or mature SP populations between WT and Kif7+/− (Figure 1C-1F).

We then examined cell surface expression of CD5, CD69 and CD24. CD5 is a negative regulator of TCR signalling and level of cell surface CD5 expression correlates with TCR signal strength [25, 26]. CD69 is an activation marker that is expressed during TCR repertoire selection following TCR signal transduction, and CD24 (HSA) is a maturation marker that is down-regulated as developing T-cells become more mature [27, 28]. DP thymocytes express high levels of CD24 and then acquire CD69 expression as a result of TCR signalling for positive selection. Newly positively-selected SP thymocytes also express high levels of CD24 and CD69, and as they mature they down-regulate both CD24 and CD69. Therefore, to determine maturation status and estimate TCR signal strength in the DP and SP populations, we measured expression of CD5, CD69 and CD24. No differences were detected in Kif7+/− DP, CD4SP and CD8SP populations compared to WT (Figure 1G).

We also examined T-cell populations in WT and Kif7+/− spleen and lymph node [29]. We found no differences in the number of cells in the spleen or LN between WT and Kif7+/−, or in the proportion or numbers of CD4 and CD8 T-cells in either tissue (Figure 1H-1M).

This analysis therefore indicates that loss of one copy of *Kif7* is dispensable for adult T-cell development.

### Kif7 regulates early thymocyte development and differentiation from DN to DP in the fetal thymus

**Figure 2:**
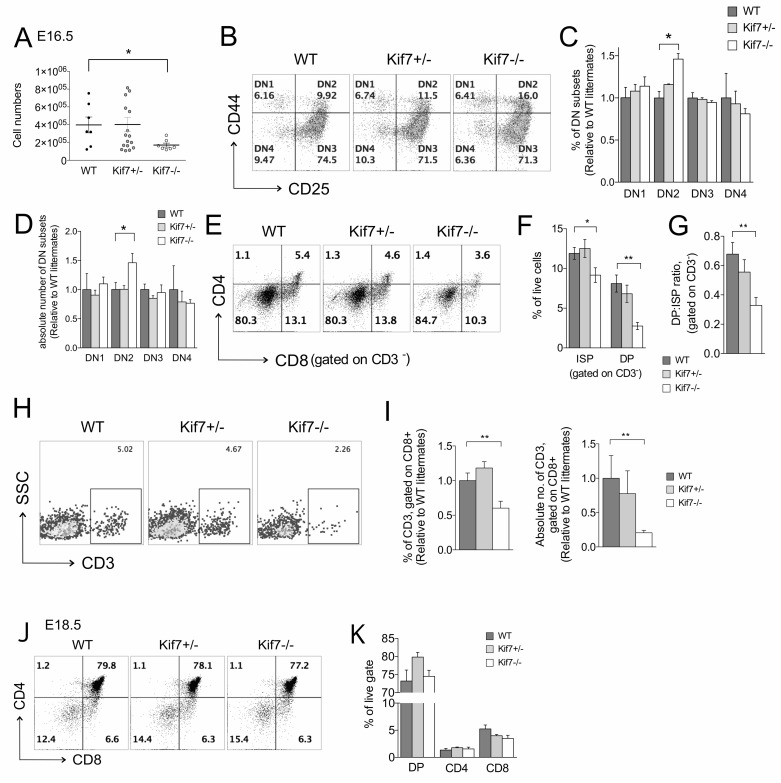
Increased DN2 population but impaired DN to DP transition in E16.5 Kif7−/− embryos In all bar charts in this figure, error bars show the standard error of the mean (SEM). **A**.-**H**. Analysis of developing thymocytes, isolated from E16.5 WT (*n* = 6), Kif7+/−(*n* = 16) and Kif7−/− (*n* = 7) fetal thymus. **A**. Scatter plot: thymocyte number in the fetal thymus. Each data point represents a single embryo. The mean for each group is indicated with a line. (*p = 0.03, WT versus Kif7−/−) **B**. Dot plots: flow cytometry profiles of DN subsets (DN1-DN4) by the surface expression of CD44 and CD25, gated on CD4-CD8- thymocytes, giving the percentage in each quadrant. **C**. Bar chart: mean relative percentage of DN1-DN4 subsets of the DN population. Each embryo was compared to its own littermates, and to allow comparison between litters for each embryo, for each subset, the relative percentage was calculated by dividing by the mean of the WT value for that litter; for DN2, *p = 0.02, WT versus Kif7−/−. **D**. Bar chart shows absolute number of cells in each DN thymocyte populations, relative to mean number of cells in that population from WT littermate embryos, in order to allow comparison between litters, where gestational age may vary by up to 12 hours; for DN2, *p = 0.045 WT versus Kif7−/−. **E**. Dot plots: flow cytometry profiles show anti-CD4 and anti-CD8 staining, gated on CD3-, to identify ISP and DP populations. The percentage of cells in each quadrant is given. **F**. Bar chart: mean percentage of ISP and DP populations (gated on CD3-); for ISP *p = 0.03; DP **p = 0.002, WT versus Kif7−/−. **G**. Bar chart: mean ratio of DP:ISP (relative to mean of WT). (**p = 0.003, WT versus Kif7−/−). **H**. Dot plots: flow cytometry of SSC and anti-CD3, gated on CD8+ cells giving the percentage of CD3+ cells. **I**. Left-hand bar chart: mean relative percentage of CD3+ cells, gated on CD8+; **p = 0.004, WT versus Kif7−/−. Right-hand bar chart: mean number of CD3+ cells (gated on CD8+), relative to mean of number from WT littermates; **p = 0.007, WT versus Kif7−/−. **J**.-**K**. Analysis of thymocytes isolated from E18.5 WT (*n* = 3), Kif7+/− (*n* = 3) and Kif7−/− (*n* = 3) littermate fetal thymus. Number of thymocytes (mean+/−SEM): WT 3.1 x10^6^ +/− 5.0 × 10^5^; Kif7+/− 3.2 × 10^6^ +/− 4.3 × 10^5^; Kif7−/− 2.8 × 10^6^ +/− 2.6 × 10^5^; there were no significant differences in the number of thymocytes. **J**. Dot plot: flow cytometry profiles of anti-CD4 and anti-CD8, giving percentage in each quadrant. **K**. Bar chart shows mean percentage of cells in DP, CD4SP and CD8SP populations.

Next, to determine the effect of Kif7-deficiency on T-cell development, we examined T-cell development in WT, Kif7+/− and Kif7−/− E16.5 fetal thymus. We analysed E16.5 because this is the day of development when DP cells first appear, and because we observed highest Kif7 expression in the DN4 subset, which are making the pre-TCR induced transition from DN3 to DP cell. Thymocyte numbers in Kif7−/− embryos were reduced significantly, with on average 50% fewer cells than in WT littermates (Figure 2A). Analysis of the DN populations revealed a significant increase in the percentage and number of DN2 cells in Kif7−/− compared to WT (Figure 2B-2D). The DN2 population contains the first cells that are committed to the T-cell lineage, and differentiation and expansion of this population is promoted by Hh signalling [3, 5, 14, 17], suggesting that Kif7 may function to negatively regulate the Hh signal at this developmental stage.

At the transition from DN3 to DP cell, the proportions of CD8ISP and DP populations were significantly reduced in Kif7−/− compared to WT littermates (Figure 2E-2F), and the ratio of DP:ISP cell was significantly reduced by 50% compared to WT (Figure 2G), indicating that the transition from ISP to DP was impaired.

Expression of cell surface CD3 is essential for TCR signalling and cell surface CD3 is up-regulated after pre-TCR signalling, as thymocytes rearrange the *TCR*α locus and express the cell surface TCR/CD3 complex. We measured cell surface expression of CD3 on CD8+ thymocytes (DP and ISP and CD8SP) and found that the proportion and number of CD3+ cells in the Kif7−/− thymus was significantly reduced compared to WT, typically to 2.26% in Kif7−/− compared to 5.02% in WT (Figure 2H-2I). In contrast, we found no significant differences in the Kif7+/− thymus compared to WT, indicating that one copy of Kif7 was sufficient to maintain homeostasis during T-cell development in the fetal thymus, as in the adult thymus.

On E18.5, the DP population in the Kif7−/− thymus had recovered and was similar to WT, indicating that DP cells could survive and accumulate (Figure 2J-2K).

### Reduced differentiation from DP to CD8SP in Kif7−/− thymus

**Figure 3:**
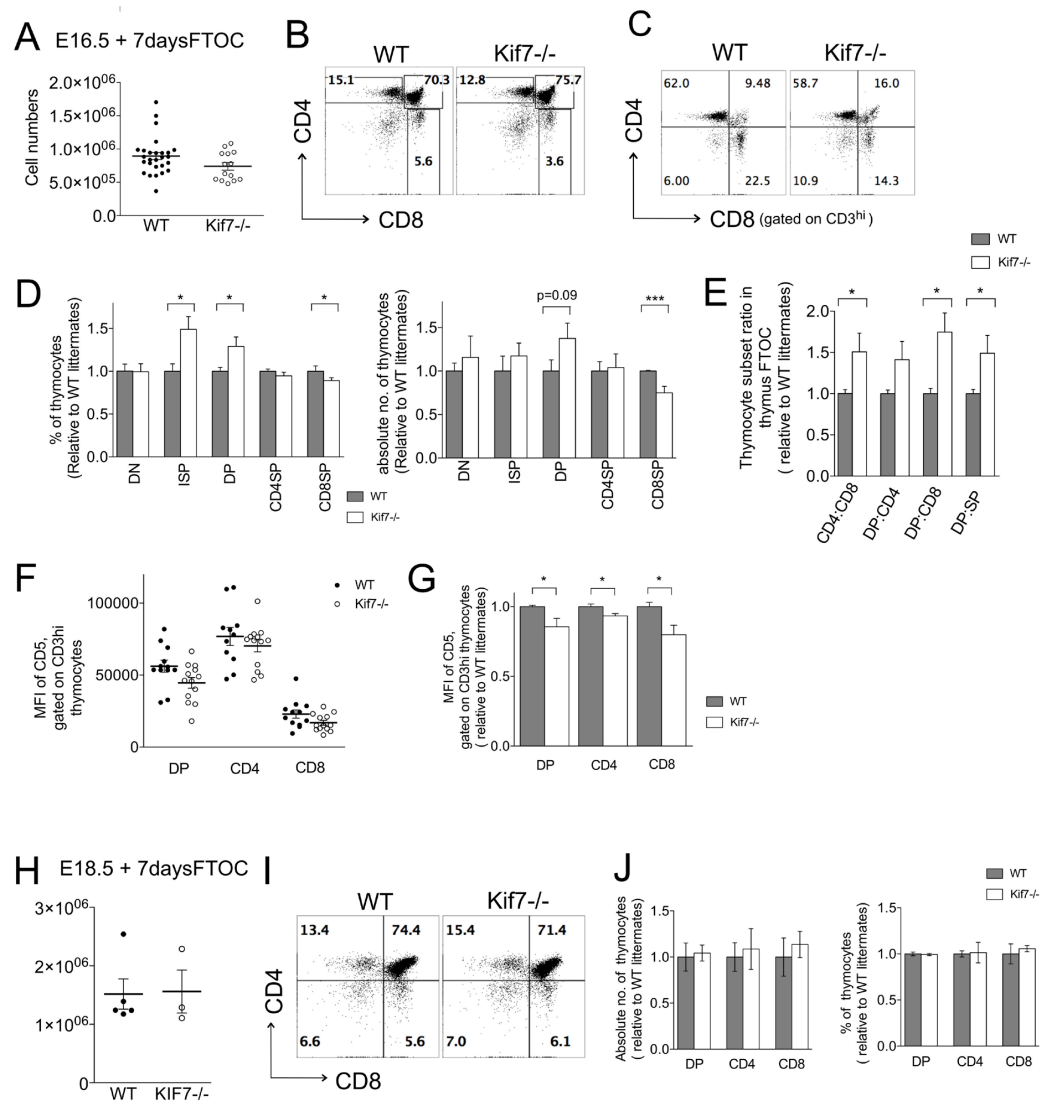
SP development in Kif7−/− FTOC In all bar charts in this figure, error bars show the standard error of the mean (SEM). **A**.-**G**. Analysis of E16.5 WT (*n* = 26) and Kif7−/− (*n* = 14) FTOC cultured for 7 days. **A**. Scatter plot: cell number in E16.5 WT and Kif7−/− FTOC after 7 days in culture. Each data point represents a single thymus. The mean for each group is indicated with a line. **B**. Dot plot: flow cytometry of anti-CD4 and anti-CD8, showing percentage of DP, CD4SP and CD8SP thymocytes. **C**. Dot plot: flow cytometry of anti-CD4 and anti-CD8, gated on CD3hi cells, giving percentage of cells in each quadrant. **D**. Left-hand bar chart shows mean relative percentages of DN, ISP, CD3hiDP, CD4SP, and CD8SP populations; for CD8ISP,*p = 0.04; for CD3hiDP, *p = 0.026; for CD8SP, *p = 0.03. Right-hand bar chart shows mean absolute number of cells in each populations (relative to mean of WT littermates); for CD8SP,***p<0.001 **E**. Bar chart: mean relative thymocyte subset ratios (ratio relative to mean of WT ratio from the same litter); CD4:CD8, *p = 0.045; DP:CD8; *p = 0.01; DP:SP, *p = 0.04. **F**. Scatter plot shows MFI of anti-CD5 staining on DP, CD4SP and CD8SP populations from WT (solid circles, *n* = 11) and Kif7−/− (open circles, *n* = 12) FTOC. Each point represents an individual thymus. Lines show mean MFI. For both WT and Kif7−/− thymus, differences between DP, CD4SP and CD8SP were significant (*p* < 0.001) **G**. Bar chart: mean of relative MFI of anti-CD5 staining (relative to mean of WT littermate MFI for that population), gated on CD3hi, in DP, CD4SP and CD8SP populations; for DP, *p = 0.04; CD4, *p = 0.01; CD8, *p = 0.02. **H**.-**J**. Analysis of E18.5 WT (*n* = 5) and Kif7−/− (*n* = 3) FTOC cultured for 7 days. **H**. Scatter plot: number of cells in the thymus, the line shows the mean. Differences were not significant. **I**. Dot plots: flow cytometry of anti-CD4 and anti-CD8, giving the percentage in each quadrant. **J**. Left-hand Bar chart shows mean relative absolute number of cells in DP, CD4SP and CD8SP populations in WT (solid bars) and Kif7−/− (open bars) (relative to mean of WT littermates). Right-hand bar chart shows relative percentage of cells in DP, CD4SP and CD8SP populations, relative to mean percentage in WT littermates.

To investigate the influence of loss of Kif7 on later stages of T-cell development, we cultured E16.5 fetal thymus organ cultures (FTOC), and compared Kif7−/− with their WT littermates. This enabled us to investigate the transition from DP to SP cell as SP cells first arise, in a more or less synchronised wave. After 7 days in FTOC, we found similar thymocyte numbers in Kif7−/− compared to WT (Figure 3A). Analysis of the thymocyte subsets showed the CD8SP population in Kif7−/− was significantly reduced, whereas the proportion of ISP cells was significantly increased (Figure 3B-3D). Following the 7-day culture period, the DP population had recovered in the Kif7−/− FTOC, again indicating that these cells were able to survive and accumulate, and consistent with the phenotype of the E18.5 thymus. The mean proportion of CD3hiDP cells increased in the Kif7−/− compared to WT, and it therefore appeared that DP cells accumulated at the transition from DP to SP, because the development of mature CD8SP cells was partially arrested.

We therefore compared the ratio of DP:CD4SP, DP:CD8SP, DP:SP and CD4SP:CD8SP in Kif7−/− FTOC from individual embryos to WT counterparts. Both DP:CD8 and DP:SP ratios in the Kif7−/− were significantly increased by 75% and 49% respectively, compared to WT (Figure 3E). Moreover, the CD4:CD8 ratio was increased by 50% in Kif7−/− FTOC (Figure 3E). The increase in the DP:CD8 and CD4:CD8 ratio in the Kif7−/− FTOC suggested that Kif7 promotes CD8-lineage differentiation. As differentiation to the CD8 lineage generally requires weaker and shorter duration of TCR signalling than differentiation to CD4-lineage, we then examined cell surface CD5, as a measure of TCR signal strength (Figure 3F-3G). We found significantly reduced CD5 expression in Kif7−/− DP compared to WT littermates. Gating on CD3hi cells showed that average MFI of CD5 was lowest on the CD8SP population, intermediate in DP cells, and highest on the CD4SP population in WT and Kif7−/− (Figure 3F). Mean MFI of anti-CD5 staining was significantly lower in all populations on Kif7−/− than on their WT counterparts, and with greater differences in DP and CD8SP than CD4SP (Figure 3F-3G).

We then cultured Kif7−/− E18.5 FTOC, in order to investigate the impact on SP populations after they first appear (Figure 3H-3J). The proportion of thymocyte populations in Kif7−/− FTOC was similar to WT, suggesting a delay rather than arrest in CD8SP differentiation.

### Kif7−/− thymocytes are refractory to modulation of external Hh signals

The Kif7-deficient embryo displays a similar phenotype to the Gli3-deficient embryo suggesting that, like Gli3, Kif7 plays a predominantly negative regulatory role in Hh signalling [8]. However, further investigations showed that Kif7 is a core modulator, which is also required for Shh activation by organizing the cilium architecture [6].

**Figure 4:**
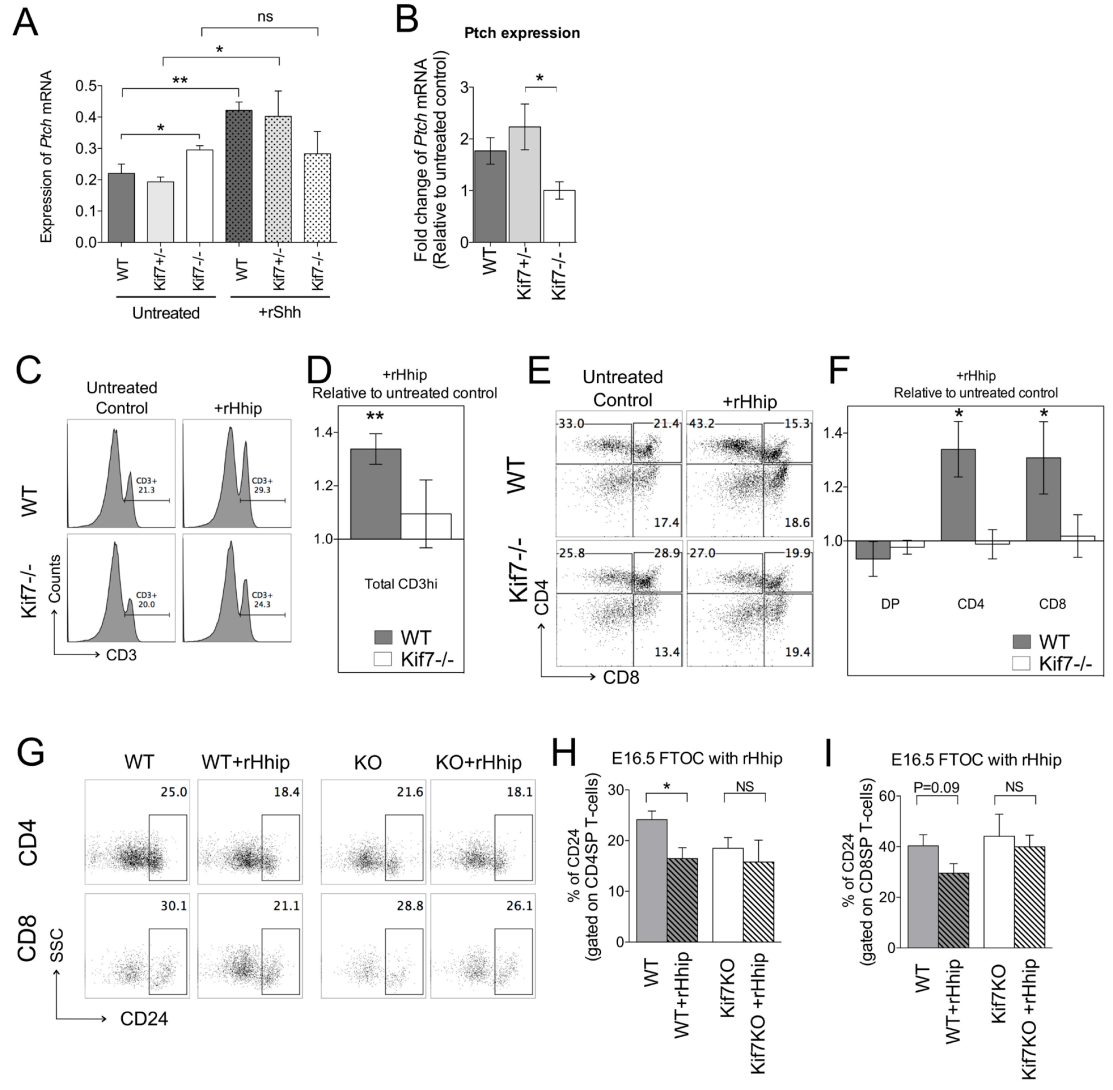
Response of Kif7−/− thymus to Shh- and Hhip-treatment In all bar charts in this figure, error bars show the standard error of the mean (SEM). E16.5 WT and Kif7−/− FTOC cultured with or without rShh or rHhip for 7 days. This experiment was carried out independently three times on different occasions (three sets of biological replicates). **A**. Transcription of *Ptch* in the E16.5 WT, Kif7+/− and Kif7−/− FTOC treated or untreated with rShh assessed by quantitative (Q) RT-PCR. The expression levels were normalized against *Hprt*. Data represent mean of the three independent experiments. (*p = 0.04, untreated WT versus untreated Kif7−/−; **p = 0.007, untreated WT versus +rShh WT; *p = 0.04, untreated Kif7+/− versus +rShh Kif7+/−; ns, not significant). **B**. Mean fold change in *Ptch* expression on rShh treatment (relative to *Ptch* expression in untreated FTOC of same genotype) in WT, Kif7+/− and Kif7−/− FTOC; *p = 0.041 for Kif7+/− versus Kif7−/−. (C-I) WT (*n* = 6) and Kif7−/− (*n* = 6) FTOC were cultured with or without rHhip treatment for 7 days. Data are from three independent experiments. **C**. Histograms show CD3 expression on untreated control and rHhip treated Kif7−/− and WT FTOC in a representative experiment, giving the percentage of cells in the positive marker. **D**. Bar chart: the relative mean percentage of CD3hi cells on rHhip treatment (relative to mean percentage of CD3hi thymocytes in untreated control FTOC of same genotype); **p = 0.008, untreated WT versus WT +rHhip. The difference between untreated Kif7−/− versus Kif7−/− +rHhip was not statistically significant. **E**. Dot plots: CD4 and CD8 staining, gated on CD3hi in WT and Kif7−/− untreated control and rHhip-treated FTOC. The percentages of CD4SP, DP and CD8SP are stated in the quadrants. **F**. The relative mean percentage, gated on CD3hi, of DP, CD4SP and CD8SP populations in rHhip-treated FTOC (relative to percentage in untreated control of same genotype) WT (solid bars) and Kif7−/− (open bars); CD4,*p = 0.04; CD8, *p = 0.04; untreated WT versus +rHhip WT. **G**. Dot plots: flow cytometry of SSC versus anti-CD24 staining on CD4SP and CD8SP thymocyte populations in WT and Kif7−/− untreated control and rHhip-treated FTOC. The percentage of cells that are CD24^hi^ is given. **H**. and **I**. Bar charts show the mean percentage of CD24^hi^ cells in untreated control or rHhip-treated WT and Kif7−/− FTOC, for (H) CD4SP cells, and (I) CD8SP cells (*p = 0.03, untreated WT versus WT+ rHhip for CD4SP).

We therefore tested if Kif7−/− thymocytes could respond to Shh treatment. We treated WT, Kif7+/− and Kif7−/− FTOC with recombinant Shh (rShh) for 7 days and assessed expression of the Hh target gene, *Ptch1* by QRT-PCR in thymocyte cell suspensions (>98% purity) prepared from the FTOC, in three independent experiments. We found that *Ptch1* expression was significantly higher in the control Kif7−/− cultures compared to WT, whereas *Ptch1* expression in Kif7+/− cultures was not significantly different from WT (Figure 4A). *Ptch1* expression was up-regulated significantly in rShh-treated WT and Kif7+/− FTOC compared to untreated control, but interestingly, *Ptch1* expression was not up-regulated in rShh-treated Kif7−/− FTOC compared to untreated Kif7−/− control (Figure 4A-4B). Thus, although Kif7−/− cells had higher levels of *Ptch1* initially, they seemed refractory or less sensitive to Hh pathway activation, suggesting that the influence of Kif7-deficiency was cell intrinsic.

We then tested the hypothesis that Kif7−/− thymocytes are refractory to modulation of external Hh signals by inhibiting the physiological Hh signal in FTOC by treatment of recombinant Hedgehog-interacting protein (rHhip), which binds and neutralises endogenous Hh protein in the cultures. Neutralisation of Hh signals in FTOC by treatment with neutralising anti-Hh monoclonal antibody has previously been shown to promote T-cell development, and increase differentiation from CD3^hi^ DP to mature SP cell and to increase the CD4SP:CD8SP ratio [19, 20]. Hhip-treatment significantly increased the proportion of CD3^hi^ thymocytes in the WT FTOC, compared to control untreated cultures (Figure 4C-4D). As expected, the proportion of CD3^hi^ thymocytes was lower in the untreated control Kif7−/− FTOC than WT, consistent with the reduction in T-cell development on E16.5. Interestingly in three independent experiments, Hhip-treatment did not significantly affect the proportion of CD3^hi^ cells in the Kif7−/− FTOC (Figure 4D). Gating on CD3^hi^ cells, the WT rHhip-treated FTOC showed a significant increase in the proportion of CD4SP and CD8SP cells, and an increase in the CD4SP:CD8SP ratio compared to WT control FTOC. Control Kif7−/− FTOC had reduced differentiation from CD3^hi^DP to CD8SP compared to WT (Figure 4E). To examine the influence of Kif7 on rHhip-treatment during the transition from DP to SP, we compared the proportion of each population in the CD3^hi^ gate from the untreated lobe to that of the rHhip-treated lobe from the same thymus, and calculated the relative increase or decrease in each population (Figure 4F). WT rHhip-treated FTOC showed significant increases in the CD4SP and CD8SP populations compared to their untreated controls, but rHhip treatment had no significant effect on the proportion of DP or mature SP populations in the Kif7−/− FTOC. Hhip-treatment reduced cell surface expression of the maturation marker CD24 on WT SP populations, indicating that not only did Hh-neutralisation increase the proportion of SP cells, but also the maturity of those cells produced. However, rHhip-treatment had no significant effect on CD24 expression in Kif7−/− FTOC (Figure 4G-4I).

Overall, these experiments indicated that T-cell development in the Kif7−/− thymus is less sensitive to modulation of the external Hh-signal than in WT thymus.

### Kif7 influences TEC

**Figure 5:**
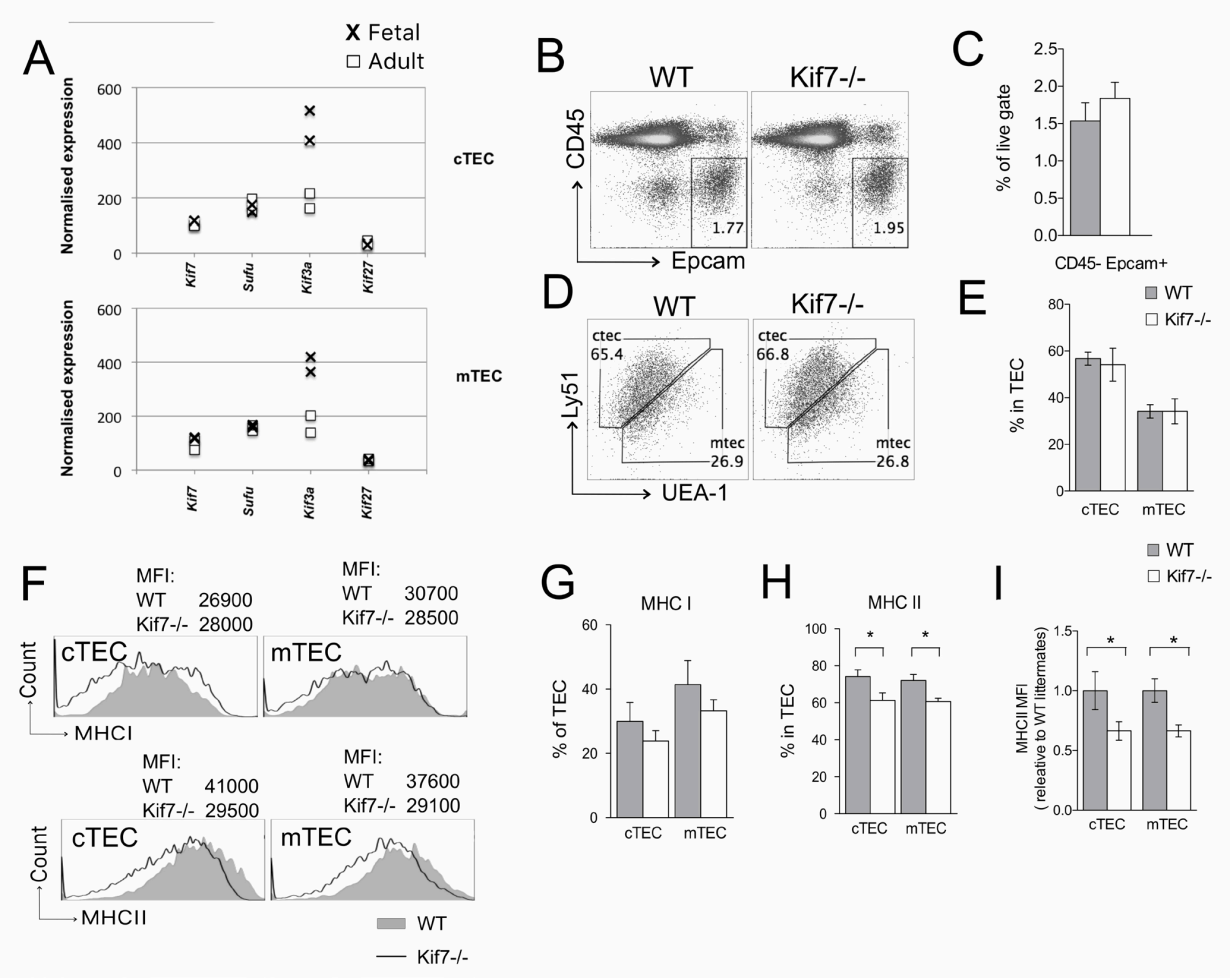
TEC population in Kif7−/− In all bar charts in this figure, error bars show the standard error of the mean (SEM). **A**. Expression of *Kif7* and related genes assessed by microarray (GSE81433) from sorted cTEC (CD45-EpCam1+Ly51+UEA-1-, upper plot) and mTEC (CD45-EpCam1+Ly51-UEA-1+, lower plot) extracted from fetal (crosses) and adult (squares) thymus. (B-I) E16.5 WT (*n* = 4) and Kif7−/− (*n* = 5) FTOC were cultured for 7 days and TEC analysed by flow cytometry. **B**. Dot plot shows anti-CD45 and anti-Epcam1 staining on WT and Kif7−/− FTOC. **C**. Bar chart: percentage of TEC (CD45-Epcam1+) cells in WT and Kif7−/−. **D**. Facs profiles and **E**. bar chart shows proportion of cTEC (Ly51+UEA-1-) and mTEC (Ly51-UEA-1+) populations in WT and Kif7−/− FTOC. **F**. Histograms: cell surface staining for MHCI (upper panel) and MHCII (lower panel) on cTEC and mTEC populations isolated from WT (solid grey) and Kif7−/− (black line), giving MFI for each population. (G-I) Bar charts show mean percentage of **G**. MHCI^hi^ cells and **H**. MHCII^hi^ cells in cTEC and mTEC populations from WT (solid bars) and Kif7−/− (open bars) fetal thymus; for MHCII, cTEC, *p = 0.048; mTEC, *p = 0.01 **I**. Bar chart: relative mean MFI (relative to mean of WT littermates) of anti-MHCII staining in cTEC and mTEC within the MHCII+ population; cTEC, *p = 0.03; mTEC, *p = 0.01.

Shh and Gli-family transcription factors are expressed in TEC, and Shh promotes TEC development and mTEC lineage choice [13, 18, 30]. We therefore examined *Kif7* expression in microarray datasets from Facs sorted cTEC and mTEC populations from fetal and adult thymus. We also assessed expression of *Sufu* and *Kif3a*, which are Hh mediators in the cilia and *Kif27*, a kinesin that is related to Kif7 [31–33]. *Kif7* and *Sufu* are expressed at similar levels in the cTEC and mTEC compartments in both the fetal and adult thymus. Interestingly, *Kif3a* is highly expressed in fetal TEC and its expression is lower in TEC from adult mice, while expression of *Kif27* was very low/below detection threshold in both fetal and adult thymus tissues (Figure 5A).

Given that Kif7 is expressed by TEC as well as thymocytes, we examined TEC populations in fetal Kif7−/− and WT littermate thymus. We cultured E16.5 FTOC for 5 days, to allow differentiation of both TEC lineages. We found no significant difference in the overall number of TEC in the thymus, defined as CD45-Epcam1+ cells, or in the ratio of mature cTEC (CD45-Epcam1+Ly51+UEA-1-) to mature mTEC (CD45-Epcam1+Ly51-UEA-1+) populations (Figure 5B-5E). In Shh−/− thymus, both cTEC and mTEC express higher levels of cell-surface MHCII than WT, whereas Gli3−/− cTEC and mTEC express lower levels of cell surface MHCII than WT [13]. We therefore compared cell surface expression of MHCI and MHCII in cTEC and mTEC from Kif7−/− and WT thymus (Figure 5F-5I). In both cTEC and mTEC populations, the proportion of cells that stained positive with anti-MHCII was higher in WT than in Kif7−/−, whereas although the average proportion of cells with high cell surface expression of MHCI was also reduced in both Kif7−/− TEC populations, this difference was not significant (Figure 5F-5I). Comparison of the level of cell surface expression (MFI) of MHCII in cTEC and mTEC populations, gated on MHCII+ cells showed that the level of MHCII expression within the positive cells was also lower in Kif7−/− cTEC and mTEC populations than in WT. Thus, in the fetal thymus, Kif7 also plays a role in TEC, and the Kif7−/− TEC populations have similar changes to Gli3−/− TEC, and opposite to Shh−/−, consistent with overall greater Hh pathway activation in Kif7−/− TEC.

### Kif7−/− T-cell development in chimeric mice

**Figure 6:**
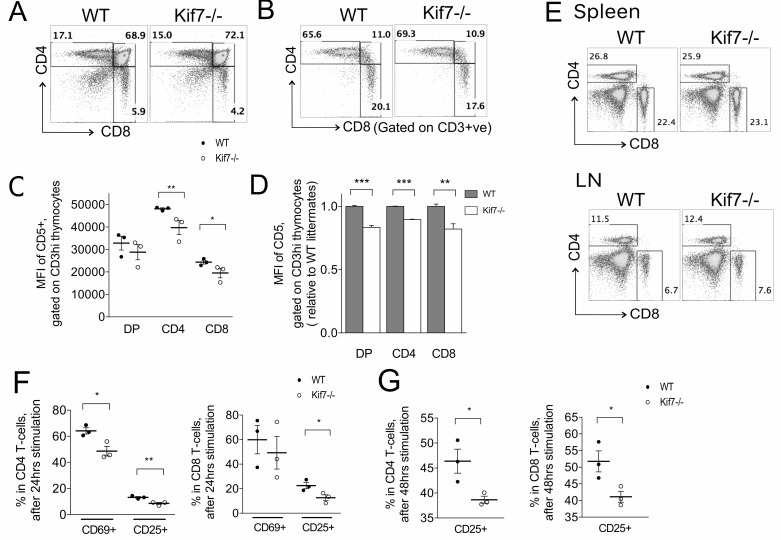
T-cell development and activation in irradiated chimeric mice In all bar charts in this figure, error bars show the standard error of the mean (SEM). **A**.-**G**. Radiation chimeras were made by transplantation of E18.5 Kif7−/− (*n* = 3) and WT (*n* = 3) fetal liver (FL) cells into sublethally irradiated Rag1−/− mice. **A**. Dot plots: anti-CD4 and anti-CD8 staining on thymocytes transplanted with WT (left) and Kif7−/− (right) fetal liver cells, giving the percentage in each quadrant. **B**. As in A, gated on CD3hi. **C**. Scatter plot: MFI of anti-CD5 staining on CD3hi DP, CD4SP and CD8 SP populations from Rag1−/− transplanted with Kif7−/− (open circles) and WT (solid circles) fetal liver cells. Each point represents a mouse. Line shows the mean. The differences between the mean between chimeras made with WT and Kif7−/− fetal liver were significant for CD4SP, **p < 0.005; and for CD8SP, *p = 0.048. **D**. Relative mean MFI of CD5 staining as in **C**; for DP and CD4SP ***p < 0.0005; for CD8SP, *p < 0.01. **E**. Dot plots show anti-CD4 and anti-CD8 staining from radiation chimeras made form WT and Kif7−/− fetal liver, on spleen (left-hand plots) and LN (right-hand plots), giving the percentage of cells in the regions shown. (F-G) Scatter plots show the percentage of CD69+ cells and CD25+ cells in the CD4 T-cell population (left-hand plot) and CD8 T-cell population (right-hand plot) when splenocytes from radiation chimeras made with WT (closed circles, *n* = 3) and with Kif7−/− (open circles, *n* = 3) were activated *in vitro* with anti-CD3 and anti-CD28 treatment, after 24 hours **F**. and 48 hours **G**. Each circles represents the reading from a culture from a different chimeric mouse. Means are shown with a line, and differences were statistically significant between WT-chimeras and Kif7−/− chimeras: At 24 hours, *p = 0.034 for CD69 on CD4 T-cells; **p = 0.009 for CD25 on CD4 T-cells; *p = 0.047 for CD25 on CD8 T-cells. At 48 hours, *p = 0.037 for CD25 on CD4 T-cells; *p = 0.041 for CD25 on CD8 T-cells.

TEC provide MHC+peptide ligands to developing T-cells for positive and negative selection of the TCR repertoire and differentiation to SP cell. Therefore, the reduction in the proportion of MHCII+ cTEC and mTEC, and the reduced density of cell surface MHCII molecules on mTEC could potentially account for those differences in T-cell development in the Kif7−/− thymus that are in processes that require TCR ligation by MHCII+peptide, such as levels of cell surface CD5 expression. To investigate this, we made radiation chimeras in which all T-cells were Kif7−/−, but TEC were Kif7+/+. We introduced Kif7−/− and Kif7+/+ fetal liver (FL) cells into sublethally irradiated adult Rag1−/− mice and measured cell surface expression of CD5 on thymocytes. Both Kif7−/− and Kif7+/+ FL cells were able to seed the thymus and expand and the proportions of thymocyte populations were similar in the adult Rag1−/− thymus reconstituted with Kif7−/− cells compared to that with WT cells (Figure 6A-6B). However, on DP, CD4SP and CD8SP cells the levels (MFI) of cell surface CD5 expression were significantly lower (Figure 6C-6D). Low cell surface CD5 expression is a marker for low TCR signal strength, and these experiments show that the reduction in cell surface CD5 expression observed in the Kif7−/− thymus is the result of cell-intrinsic Kif7-deficiency in T-cells, rather than the result of lower expression of MHC+peptide ligands for TCR on developing thymocytes.

In the spleen and LN of the radiation chimeric mice, the proportions of CD4 and CD8 T-cells were similar between those reconstituted by WT or Kif7−/− FL (Figure 6E). As CD5 expression correlates with TCR signal strength we tested whether Kif7−/− T-cells activated normally on ligation of CD3 and CD28. We activated splenocytes from WT and Kif7−/− with anti-CD3 and anti-CD28 monoclonal antibodies, and compared the levels of induction of activation markers CD69 and CD25. CD69 is an early activation marker, and at 24 hours after activation, its expression was significantly lower on Kif7−/− CD4 T-cells than on their WT counterparts (Figure 6F). During T-cell activation, cell surface CD25 expression occurs after the increase in CD69 expression. At 24 hours, the percentage of cells that expressed cell surface CD25 was significantly lower in Kif7−/− CD4 T-cells and in Kif7−/− CD8 T-cells than in their WT counterparts (Figure 6F). This continued at 48 hours after activation, in both CD4 and CD8 T-cell populations (Figure 6G). Thus, T-cell intrinsic Kif7-deficiency decreased the ability of T-cells to activate in response to CD3 and CD28 ligation.

## DISCUSSION

Here we showed that Kif7 is required for normal T-cell development and MHC expression on TEC in the fetal thymus. On E16.5, Kif7-deficiency increased the DN2 population, but led to reduced differentiation from DN to DP cell. Examination of the transition from DP to SP cell, also showed delayed differentiation to CD8SP, and reduced cell surface CD5 expression, indicative of defective positive selection. Interestingly, Kif7-deficiency also impacted on TEC, and led to lower cell surface MHCII expression on mTEC and cTEC.

Overall, these changes are consistent with the Kif7−/− thymus having a higher level of Hh pathway activation, and indeed *Ptch1* expression was higher in Kif7−/− thymocytes than WT, with Kif7 thus acting as a negative regulator of Hh pathway activation. In fact, at early stages of T-cell development, Kif7-deficiency led to the opposite phenotype to deficiency in Shh and positive regulators of the pathway, as Smo-, Gli2- and Shh-deficiency all decrease the DN2 population [5, 14, 17]. At the pre-TCR dependent transition to DP cell, Kif7-deficiency has a similar impact to Gli3-deficiency, with slower differentiation to DP cell, but the opposite effect to Shh-deficiency and Gli2-deficiency [17, 34]. Likewise, Kif7-deficient TEC had a similar phenotype to Gli3-deficient TEC, and the opposite phenotype to Shh-deficient TEC, indicative of increased Hh pathway activation [13].

Interestingly, the Kif7-deficient thymus was less sensitive to addition of rShh than WT, and T-cell development was refractory to the effects of neutralisation of Hh proteins. In WT thymus neutralisation of Hh proteins promoted T-cell development: it increased the proportion of CD3hi cells, and increased maturation of both SP populations, in addition to its previously described effect to increase differentiation from CD3hiDP cell to CD4SP and CD8SP [20]. In contrast, neutralisation of Hh proteins in the Kif7−/− thymus had no impact on differentiation or maturation of the SP populations, indicating that Kif7 is required for interpretation of changes in the Hh signal by developing T-cells.

At the transition from DP to SP cell, T-cell development in the Kif7-deficient thymus seems to diverge from simply being the opposite of Shh-deficiency or Gli2-deficiency. Deletion of both Shh and Gli2 lead to increased differentiation from DP to SP and an increase in the CD4SP:CD8SP ratio, which is indicative of higher TCR signal strength [20, 35]. In the Kif7-deficient thymus, however, the differentiation of the CD8SP population was delayed, suggesting a specific role for Kif7 in this lineage.

In ciliated cells, Kif7 is believed to regulate Gli activity by controlling the structure of the primary cilium [6]. However, T-cells lack primary cilia, and ciliary proteins are located in the immune synapse [23]. We found that cell surface CD5 expression was reduced in Kif7−/− thymocytes, in the fetal thymus and in FTOC, and this reduction was the result of thymocyte-intrinsic loss of Kif7. Levels of cell surface CD5 expression correlate with the strength of TCR signal transduction, and Shh-treatment also lowers CD5 expression on thymocytes, and lowers TCR signal strength in mature T-cells [19–21]. Therefore, the fact that Kif7-deficiency led to reduced CD5 expression points to a possible link between the machinery of Hh and TCR signalling in T-cells, and to a role for Kif7 in the immune synapse. Interestingly, Kif7−/− T-cells from radiation chimeric mice were less able to activate in response to CD3 and CD28 ligation than their WT counterparts, confirming a T-cell intrinsic role for Kif7 in TCR signal transduction. Hh pathway activation has previously been shown to modulate T-cell activation and TCR signal strength [20, 21, 36]. In the future, it will be important to investigate if the role of Kif7 in T-cell activation is a result of increased Hh pathway activation or if it is an Hh-independent function of Kif7 in TCR signalling.

## MATERIALS AND METHODS

### Mice

*Kif7+/−* mice [8] were purchased from the Mutant Mouse Resource Research Center (MMRRC); Rag1−/− mice from Jackson Labs; C57BL/6 mice from Harlan. Genotyping of *Kif7* mutant mice by PCR was performed as described [37] with primers *neo* (5′-GCAGCGCATCGCCTTCTATCG-3′), exon 2 (5′-GGCGGGACCGACACTTTGGG-3′), and intron 2 (5′-CACCTGACATGGAGTGCTGACC-3′), generating a 302 bp product in WT and 197 bp product in knockout allele.

To make chimeric mice, fetal liver cells from Kif7+/+ and Kif7−/− E18.5 embryos were injected intraperitoneally into sublethally irradiated Rag1−/− mice and analysed 10-12 weeks later.

Mice were bred and maintained on a C57BL/6 background at UCL under UK Home Office regulations.

### Cell sorting

Thymocytes from 6-8 week-old C57BL/6 WT mice were stained with anti-CD25, anti-CD44, anti-CD3, anti-CD4 and anti-CD8 and sorted on a Modular Flow Cytometer (MoFlo; Cytomation, Fort Collins, CO) at ICH flow cytometry facility to obtain DN1 to DN4, DP and SP populations.

### Quantitative RT-PCR

RNA was extracted from the sorted thymocytes using Absolutely RNA miniprep kit (Stratagene, La Jolla, CA), and cDNA synthesized with Superscript II (Invitrogen, Carlsbad, CA). cDNA samples were analyzed in triplicate by quantitative PCR on an iCycler (Bio-Rad) using SYBR Green Supermix (Bio-Rad) according to manufacturer's instruction. *Kif7* and *Hprt* primers were purchased from Quantitect Primer Assays (Qiagen) and *Ptch1* primers were as described [37].

### Flow cytometry and antibodies

Cell suspensions were prepared by crushing tissue between frosted glass slides. Cells were stained as described [37], using combinations of directly conjugated antibodies: anti-CD3, anti-CD4, anti-CD5, anti-CD8, anti-CD24, anti-CD25, anti-CD44, anti-CD45, anti-CD69, anti-MHCI, and anti-MHCII (Ebioscience).

Samples were washed in staining buffer prior to acquisition on an Accuri C6 (Becton Dickinson) and analysed using Flowjo 10.1r5 (Tree Star, US). Live cells were gated by SSC and FSC, gating on singlets using Flowjo (FSC-A v FSC-H).

### Fetal thymus organ culture (FTOC)

FTOC was cultured for 7 days using E16.5 or E18.5 fetal thymus as described [38]. For experiments shown in Figure 5, one lobe of each E16.5 WT or Kif7−/− thymus was cultured untreated (control) and the other lobe cultured in the presence or absence of rShh or rHhip as described [13, 19].

### T-cell activation experiments

Splenocytes were isolated and stimulated with soluble 0.01μg/ml of anti-CD3 and anti-CD28 as described [20]. Cells were harvested at 24 and 48 hours and analysed by flow cytometry for cell surface markers of activation.

### Isolation and staining of thymic epithelial cells (TEC)

TEC were isolated as described [13] from E16.5 FTOC cultured for 5 days to allow differentiation of TEC lineages. After isolation, cells were stained with anti-CD45^APCcy7^ as a haematopoitic lineage marker and anti-Epcam1^PEcy7^, to identify the total TEC population (CD45-Epcam1+). Identification of mTEC and cTEC populations were identified as staining positive with anti-UEA-1^Fitc^ and anti-Ly51^PE^, respectively. Samples were acquired on a LSR-II (Becton Dickinson) and analysed using Flowjo 10.1r5 (Tree Star, US).

### Microarray and data analysis

Microarray analysis was carried out as described [38] on RNA from Facs sorted cTEC and mTEC from fetal and adult WT C57BL/6 mice [18]. TEC populations were isolated and sorted as described [13]. The data analysis was carried out as described [38]. The data are publicly available in http://www.ncbi.nlm.nih.gov/. GEO reference: GSE81433.

### Statistical analysis

Statistical analysis was performed using unpaired two-tailed t-tests or ANOVA and probabilities considered significant if P≤0.05 (*), P≤0.01 (**) and P≤0.001 (***). All experiments were carried out at least three times (independent experiments with biological replicates). Error bars represent SEM.

### Data availability

The microarray data are publicly available in http://www.ncbi.nlm.nih.gov/. GEO reference: GSE81433.
